# Pollution and Tuberculosis: Outdoor Sources

**DOI:** 10.1371/journal.pmed.0040142

**Published:** 2007-03-27

**Authors:** Aaron Cohen, Sumi Mehta

The meta-analysis by Lin and colleagues [1] is important for two reasons. It evaluates the evidence concerning exposure to combustion-derived air pollution and tuberculosis (TB), and it quantifies the risk of TB associated with three important sources of exposure: tobacco smoking, environmental tobacco smoke, and indoor burning of solid fuels. Their analysis invites speculation about the possible role of another combustion source: outdoor urban air pollution, a growing problem in developing countries where the burden of disease from TB is greatest.

Combustion-source air pollution affects resistance to infection via effects on airway resistance, epithelial permeability, and macrophage function [2]. Studies suggest a specific role for fine particles less than 2.5 micrometers in diameter (PM2.5) [3]. PM-associated transition metals, e.g., iron, are thought to produce oxidative stress in the lung, hypothesized to be a common factor in a range of adverse effects [4], and have been associated with altered host defenses in rats [5]. As Lin notes, the amount of iron in the lung has also been hypothesized to adversely affect the progression of TB [6,7].

Air pollution from outdoor sources, such as motor vehicles, industry, and neighborhood-level solid waste burning, is associated with increased morbidity and mortality from respiratory infections in children and adults [8,9]. Only one study of outdoor air pollution and tuberculosis has been reported in the peer-reviewed literature to date [10], but one might speculate that outdoor air pollution would have a similar impact on TB infection and/or progression of disease via the mechanisms described above.

People in urban areas of developing countries are exposed to the highest levels of outdoor air pollution in the world, which each year impose an estimated burden of hundreds of thousands of deaths and millions of years of healthy life lost from cardiovascular disease, selected respiratory diseases, and lung cancer [11]. TB was not considered due to lack of evidence, so these estimates assume that outdoor air pollution plays no role. If, however, air pollution exposure increases the risk of infection, illness, or death from TB, then the attributable burden of disease would be even greater.

Environmental policy in developing countries should be informed by the best and most complete information on the health effects of air pollution. New research efforts should address health outcomes of regional relevance, such as TB and childhood respiratory illness. Since TB is endemic in many developing countries, even a small increase in risk could translate into a large attributable burden. Research on outdoor air pollution and TB seems warranted.
